# Trends in e-cigarette use among adolescents in Malaysia, 2016–2022: A secondary dataset analysis of national surveys

**DOI:** 10.18332/tid/219767

**Published:** 2026-05-26

**Authors:** Muhammad Fadhli Mohd Yusoff, Thamil Arasu Saminathan, Tania Gayle Robert Lourdes, Mohd Ruhaizie Riyadzi, Hamizatul Akmal Abd Hamid

**Affiliations:** 1 Institute for Public Health, National Institutes of Health, Ministry of Health, Shah Alam, Malaysia

**Keywords:** electronic cigarette, e-cigarette, trend, adolescent, Malaysia

## Abstract

**INTRODUCTION:**

E-cigarette use among adolescents has increased in recent years, including in Malaysia. Monitoring changes in use is important to inform the prevention of nicotine addiction. This study examines changes in adolescent e-cigarette use in Malaysia and associated sociodemographic factors from 2016 to 2022.

**METHODS:**

This study was a secondary analysis of data from three national school-based health surveys in Malaysia: Tobacco and E-Cigarette Survey among Malaysian Adolescents (TECMA) 2016, and National Health and Morbidity Survey (NHMS) 2017 and 2022. The analysis included school-going adolescents aged 13–17 years. Complex sample analysis was used to estimate prevalence, and multivariable logistic regression using NHMS 2017 and NHMS 2022 examined associated factors. All surveys used a two-stage stratified cluster sampling design and a questionnaire adapted from the Global Youth Tobacco Survey.

**RESULTS:**

A total of 13162, 27497, and 33523 respondents participated in TECMA 2016, NHMS 2017, and NHMS 2022. The prevalence of current e-cigarette use among adolescents aged 13–17 years was 12.0% (95% CI: 10.1–14.3), 9.8% (95% CI: 9.0–10.8), and 14.9% (95% CI: 13.7–16.1) in 2016, 2017, and 2022, respectively. Multivariable logistic regression showed that the adjusted odds ratio (AOR) of e-cigarette use increased from 2017 to 2022 among adolescents who smoke from 15.25 (95% CI: 12.62–18.43) to 56.29 (95% CI: 46.76–67.77), among Malays from 1.78 (95% CI: 1.28–2.48) to 3.71 (95% CI: 3.02–4.57), and among students whose parents smoke from 1.0 (95% CI: 0.85–1.19) to 1.32 (95% CI: 1.16–1.49). The association with parental e-cigarette use decreased from 3.78 (95% CI: 3.00–4.77) to 1.64 (95% CI : 1.42–1.89).

**CONCLUSIONS:**

E-cigarette use among adolescents has increased in Malaysia. While associated factors remain generally consistent, their strength has changed across survey waves. These findings highlight the need for continued public health efforts and appropriate regulatory strategies to address e-cigarette use among adolescents in Malaysia.

## INTRODUCTION

Electronic cigarettes (e-cigarettes), also known as ‘e-cigs’ and ‘vapes’, have become a new threat to public health since their introduction into the market in the past two decades^1^. E-cigarettes are battery-powered devices that allow users to inhale nicotine into the lungs through vapor^2^. Besides nicotine, e-cigarette aerosol can also contain other substances such as cancer-causing chemicals, volatile organic compounds, and heavy metals that can harm health^3,4^.

As e-cigarette use is relatively new in the population, the long-term effects of its use are still being studied, though growing evidence suggests several potential health risks^5,6^. Prolonged nicotine exposure can lead to addiction, and studies have also raised concerns about the potential for e-cigarettes to act as a gateway to traditional cigarette smoking, particularly among adolescents^7^. Research also indicates that long-term e-cigarette use may contribute to cardiovascular diseases by damaging blood vessels and promoting inflammation^6^. Chronic exposure to e-cigarette aerosol has been linked to measurable airway inflammation and biological alterations in human lung tissue, including increases in inflammatory markers^8^.

The use of e-cigarettes, especially among adolescents, has risen substantially in recent years^2,9^. In Malaysia, the first national survey conducted in 2016 reported that 9.1% of adolescents aged 10–19 years were current e-cigarette users^10^. Evidence from previous reviews indicates that individual factors such as older age, male sex, and concurrent use of conventional cigarettes are associated with a higher likelihood of e-cigarette use among adolescents^11^. Besides individual determinants, peer influence^11^ and parental factors such as parental use of tobacco or nicotine products, have also been consistently linked to adolescent e-cigarette uptake^12^. Monitoring the prevalence and trends of e-cigarette use is therefore critical for informing public health strategies aimed at preventing nicotine addiction among young people. This study examines changes in the prevalence of e-cigarette use among adolescents in Malaysia across three national surveys conducted between 2016 and 2022.

## METHODS

This study is a secondary analysis of three nationally representative school-based surveys conducted among adolescents in Malaysia: Tobacco and E-cigarette Survey among Malaysian Adolescents (TECMA) 2016, National Health and Morbidity Survey (NHMS) 2017, and NHMS 2022. The NHMS 2017 and NHMS 2022 had a similar target population, which consisted of adolescents aged 13–17 years (Forms 1–5, corresponding to lower and upper secondary school levels), while the target population for TECMA was adolescents aged 10–19 years. In studying the trend, data on adolescents aged 13–17 years were extracted from the TECMA 2016 dataset and reanalyzed to make it comparable to the NHMS data.

The surveys used a two-stage stratified cluster sampling design to select a representative sample of school-going adolescents in Malaysia. Stratification was according to the states in Malaysia. Two-stage sampling was performed within each state, with schools as the primary sampling unit (PSU) and classes within the selected schools as the secondary sampling unit (SSU). Schools from each stratum were randomly selected via a probability-proportionate-to-size (PPS) method, and classes were randomly selected from each selected school. All students from the selected classes were included in the survey.

Ethical approval was obtained from the Medical Research and Ethics Committee (MREC), Ministry of Health Malaysia [TECMA 2016 (NMRR-16-108-28789), NHMS 2017 (NMRR-16-698-30042), NHMS 2022 (NMRR-21-157-58261)]. Informed consent was obtained from all subjects and, for subjects under 18 years, from a parent and/or legal guardian. All methods were carried out in accordance with relevant guidelines and regulations.

### Questionnaire

All three surveys (TECMA 2016, NHMS 2017, and NHMS 2022) used a structured questionnaire that was adapted from the Global Youth Tobacco Survey Questionnaire^13^. The questionnaire consists of questions on tobacco smoking, the type of tobacco products used, and e-cigarette use. Additionally, the questionnaire also included questions on sociodemographic characteristics such as gender, age, and ethnicity. As for e-cigarette use, adolescents who answered ‘Yes’ to the question ‘During the past 30 days, did you use an e-cigarette or vape?’, were defined as current e-cigarette users.

### Data collection

All three surveys used a similar method in collecting the data. The selected schools were approached to get permission to participate in the study. The data collection team met the students in their classrooms and explained the study. Their anonymity and confidentiality were assured, and their participation in the study was voluntary. Informed consent was obtained from the respondents’ parents or guardians prior to data collection, in addition to the respondents’ assent. Data was collected by a self-administered questionnaire.

### Data analysis

Data were cleaned, and variables were categorized according to predefined definitions. Descriptive analyses were conducted to summarize respondents’ characteristics. Frequencies and percentages are used to describe categorical variables, and prevalence estimates of e-cigarette use are presented with corresponding 95% confidence intervals (CIs), accounting for the complex sampling design and survey weights to ensure national representativeness. Differences in the distribution of sociodemographic characteristics across survey waves were assessed using appropriate statistical tests for complex survey data.

Multivariable logistic regression analysis was performed to examine factors associated with current e-cigarette use among adolescents using data from NHMS 2017 and NHMS 2022. Regression analyses were conducted separately for each survey wave to allow comparison of associated factors across surveys with similar methodology. The dependent variable was current e-cigarette use. Independent variables included sex, age group (school form), ethnicity, smoking status, parental smoking status, and parental e-cigarette use. Age categories were derived from school forms 1–5, which correspond approximately to ages 13–17 years in the Malaysian education system. All possible two-way interactions between independent variables were assessed during model development. The model fit was assessed using Nagelkerke R^2^ and a classification table. All analyses were conducted using SPSS version 28 with the Complex Samples module^13^.

## RESULTS

A total of 13162, 27497, and 33523 respondents participated in TECMA 2016, NHMS 2017, and NHMS 2022, with response rates of 88.7%, 89.2%, and 89.4%, respectively. Out of 13162 respondents, 8924 data of individuals aged 13–17 years were extracted from TECMA 2016 for this study. Table 1 shows the sociodemographic characteristics of the respondents from each survey in this study.

**Table 1:** Sociodemographic characteristics of school-going adolescents aged 13–17 years in Malaysia, by survey wave (TECMA 2016, NHMS 2017, NHMS 2022), 2016–2022

*Variables*	*2016* *(N=8924)*		*2017* *(N=27497)*		*2022* *(N=33523)*	
	*n*	*%*	*n*	*%*	*n*	*%*
**Location**						
Urban	5062	56.7	15899	57.8	28165	84
Rural	3862	43.3	11598	42.2	5358	16
**Sex**						
Male	4366	48.9	13135	47.8	15493	46.2
Female	4558	51.1	14362	52.2	18030	53.8
**Age** (years)						
13 (Form 1)	1636	18.3	5704	20.8	7216	21.5
14 (Form 2)	1838	20.6	5501	20	6902	20.6
15 (Form 3)	1813	20.3	5837	21.2	6460	19.3
16 (Form 4)	2195	24.6	5532	20.1	6756	20.1
17 (Form 5)	1442	16.2	4923	17.9	6189	18.5
**Ethnicity**						
Malays	6133	68.7	18713	68.1	23125	67.2
Chinese	1310	14.7	4100	14.9	5985	17.4
Indians	537	6	1428	5.2	1556	4.5
Other	943	10.6	3256	11.8	3757	10.9

The surveys showed that 12.0%, 9.8%, and 14.9% of adolescents aged 13–17 years were current e-cigarette users in 2016, 2017, and 2022, respectively, corresponding to approximately 238000, 211000, and 307000 adolescents nationwide based on weighted population estimates. The prevalence of e-cigarette users was consistently higher in males (15.5–23.5%) compared to females (2.8–6.2%), rural dwellers (11.2–20.8%) compared to urban dwellers (8.8–13.5%), and increased with age in all three surveys (Table 2).

**Table 2:** Prevalence of current e-cigarette user by sociodemographic characteristics of school-going adolescents aged 13–17 years in Malaysia by survey wave (TECMA 2016, NHMS 2017, NHMS 2022) 2016–2022

*Variables*	*2016 (N=8924)*					*2017 (N=27497)*					*2022 (N=33523)*				
	*Total n*	*Estimated population*	*Prevalence (%)*	*95% CI*		*Total n*	*Estimated population*	*Prevalence (%)*	*95% CI*		*Total n*	*Estimated population*	*Prevalence (%)*	*95% CI*	
				*Lower*	*Upper*				*Lower*	*Upper*				*Lower*	*Upper*
**Overall**	860	237639	12	10.1	14.3	2547	211084	9.8	9	10.8	4640	307109	14.9	13.7	16.1
**Location**															
Urban	481	82907	11.3	9.3	13.6	1365	106181	8.8	7.7	10	3650	226132	13.5	12.2	14.9
Rural	379	154732	12.5	9.7	15.9	1182	104903	11.2	9.9	12.7	990	80977	20.8	18.1	23.7
**Sex**															
Male	743	204597	22.1	19.1	25.4	2188	180620	17	15.5	18.5	3582	242849	23.5	21.6	25.6
Female	117	33042	3.1	1.9	5.3	359	30464	2.8	2.4	3.3	1058	64260	6.2	5.7	6.8
**Age** (years)															
13 (Form 1)	103	39690	9.3	5.3	15.9	423	38583	8.6	6.8	10.7	708	47928	10.7	9.3	12.3
14 (Form 2)	174	37794	10.7	8.3	13.7	507	40545	9.5	8.1	11.1	806	55129	12.8	11.4	14.3
15 (Form 3)	162	63199	13.2	9.1	18.8	553	43446	10.1	8.4	12.1	858	63398	15.2	13.6	17
16 (Form 4)	274	60065	13.1	9.6	17.6	562	44040	10.6	9.1	12.5	1179	67708	17.3	14.7	20.2
17 (Form 5)	147	36891	14.1	9.5	20.4	502	44470	10.5	8.7	12.7	1089	72946	19.1	16.8	21.8
**Ethnicity**															
Malays	684	179492	14.8	12.2	17.8	1797	138875	10.3	9.3	11.3	3477	220305	16.9	15.5	18.5
Chinese	32	9302	2.9	1.6	5.2	166	16176	4.5	3.5	5.8	254	19530	5.2	4.3	6.3
Indians	31	8974	6.8	4.4	10.5	133	16264	10.9	7.3	16.1	170	13165	10.7	8.5	13.4
Other	113	39871	13.1	9.1	18.4	451	39769	14	12.1	16.2	739	54109	20.2	18.1	22.5
**Smoking status**															
Smoker	588	169492	54.5	49.4	59.5	1859	155595	45.5	43.3	47.8	2386	157892	84.5	82.9	86
Non-smoker	272	68147	4.1	3.4	4.9	688	55489	3.1	2.7	3.5	2254	149217	7.9	7.2	8.7
Parents smoking status*															
Smoker						1313	108616	12.6	11.3	13.9	2406	160274	18.9	17.5	20.5
Non-smoker						914	76923	6.9	6.1	7.7	1843	122525	11.1	10	12.2
Parents ecig use*															
Yes						715	59433	21.5	18.8	24.5	1401	90231	22	20.4	23.6
No						1330	110955	6.8	6.1	7.6	2794	187984	12.2	11.1	13.4

* These variables were not included in TECMA 2016. ecig: e-cigarette.

Multivariable logistic regression analysis of NHMS 2017 and NHMS 2022 data revealed that, after controlling for gender, age, ethnicity, parents’ smoking status, and parents’ e-cigarette use, the adjusted odds ratio (AOR) of e-cigarette use showed a substantial increase from 2017 to 2022 among adolescents who smoke (from 15.25 to 56.29) and among Malays (from 1.78 to 3.71) and the ‘other ethnicities’ (from 2.05 to 3.83). The AOR also increased with age (school form) and among students whose parents smoke. However, the association between adolescents’ e-cigarette use and their parents’ e-cigarette use decreased, with the AOR decreasing from 3.78 to 1.64 (Table 3).

**Table 3:** Comparison of factors associated with current e-cigarette use among school-going adolescents aged 13–17 years in Malaysia using complex sample multivariable logistic regression, by survey wave (NHMS 2017, NHMS 2022)

*Variables*	*2017 (N=27497)*			*2022 (N=33523)*		
	*AOR*	*95% CI*		*AOR*	*95% CI*	
		*Lower*	*Upper*		*Lower*	*Upper*
**Sex** (ref. female)						
Male	4.4	3.53	5.48	3.81	3.37	4.3
**Age** (years) (ref. 13, Form 1)						
14 (Form 2)	1.19	0.9	1.56	1.3	1.02	1.66
15 (Form 3)	1.14	0.81	1.6	1.83	1.46	2.29
16 (Form 4)	1.27	0.97	1.65	2.35	1.79	3.08
17 (Form 5)	1.34	0.95	1.89	2.27	1.72	3.01
**Ethnicity** (ref. Chinese)						
Malays	1.78	1.28	2.48	3.71	3.02	4.57
Indians	2.28	1.39	3.75	2.25	1.59	3.19
Other	2.05	1.41	2.97	3.83	3.02	4.87
**Smoking status** (ref. non-smoker)						
Smoker	15.25	12.62	18.43	56.29	46.76	67.77
**Parents smoking status** (ref. non-smoker)						
Smoker	1	0.85	1.19	1.32	1.16	1.49
Parents ecig use (ref. no)						
Yes	3.78	3	4.77	1.64	1.42	1.89

AOR: adjusted odds ratio. Nagelkerke R^2^=0.379 for NHMS 2017 model, and R^2^=0.466 for NHMS 2022 model. The overall percentage of correct classification between predicted and observed outcomes was 92.2% for NHMS 2017 model and 91.7% for NHMS 2022 model.

## DISCUSSION

This study describes changes in the prevalence of e-cigarette use among adolescents across three national school-based health surveys conducted between 2016 and 2022, conducted in Malaysia. Although the prevalence fluctuated over the years, there was an overall increase during this period, with higher rates observed across all sociodemographic groups in 2022 compared to the earlier surveys in 2016 and 2017. This rising trend is concerning, as e-cigarette use may expose adolescents to nicotine, potentially leading to addiction and increasing the likelihood of cigarette smoking initiation among non-smokers^14,15^. The prevalence of e-cigarette use also increased in the US from 0.6% in 2011 to 4.9% in 2018 among middle school students and from 1.5% to 20.8% among high school students^16^. Based on the Global Youth Tobacco Survey from 2012 to 2020, 7 of 10 countries with 2 waves of data showed a significant increase in the adjusted prevalence of e-cigarette use, i.e. Georgia (from 5.7% to 12.2%), Italy (from 7.8% to 17.3%), Latvia (from 9.7% to 18.3), Albania (from 5.5% to 9.2%), Nicaragua (from 5.2% to 8.5%), Paraguay (from 3.6% to 12.5%), and Peru (from 2.2% to 6.6%)^17^. However, the adjusted prevalence of e-cigarette use had decreased in Iraq from 10.8% to 7.1%^17^. This overall increasing trend could be attributed to several factors, including aggressive and appealing characteristics of e-cigarette marketing targeting young people^18,19^, perceptions that e-cigarettes are less harmful than traditional cigarettes^18,20^, along with social influences and curiosity^19,21^.

Alarmingly, the prevalence of e-cigarette use among adolescents in Malaysia was higher than the global average of 9.2%^22^ and ranked among the highest in Asian countries^21^. Other countries that reported high adolescent e-cigarette use included Canada, with a national average of 31.4%^23^, Poland 17.1%^23^, Italy 17.5%^16^, Latvia 18.0%^16^, and United Kingdom 13.3%^23^. In Southeast Asia, the Philippines and Indonesia also reported high prevalence rates of current e-cigarette use, at 14.1%^21^ and 11.8%, respectively^24^. The increase in e-cigarette use observed in 2022 should also be interpreted in the context of Malaysia’s regulatory environment during the study period. Prior to recent legislative efforts, e-cigarettes were subject to relatively limited regulation, which may have contributed to increased availability, marketing exposure, and uptake among adolescents.

In this study, among adolescents in Malaysia, being a current smoker is a very strong determinant of ecigarette use: the NHMS 2022 found an AOR of 56.29 for ecigarette use among adolescents who smoke. This represents a significant increase compared to NHMS 2017, where an AOR of 15.25 for current smokers versus non-smokers was reported. The extremely high adjusted odds ratio observed among current cigarette smokers likely reflects strong clustering of nicotine-use behaviors among adolescents in 2022. Youth who already smoke cigarettes may be more willing to experiment with alternative nicotine products such as e-cigarettes. In addition, overlapping behavioral and social risk factors may contribute to concurrent use of multiple nicotine products among adolescents. The large magnitude of the association should be interpreted cautiously, as cigarette smoking and e-cigarette use represent closely related nicotine-use behaviors and may occur concurrently among adolescents^25,26^. Comparatively, in other Southeast Asian countries and more broadly, smoking status is also a strong correlate of ecigarette use, though the odds tend to be lower than Malaysia’s latest estimate. In Thailand, a study of seventhgrade students found that current cigarette smoking was associated with current ecigarette use, with an AOR of about 4.28^27^. Globally, the 2012–2019 Global Youth Tobacco Survey data show that youth who smoke cigarettes had an AOR of about 7.18 for ecigarette use compared to nonsmokers^22^.

In Malaysia, consistent with the pattern observed for conventional cigarette smoking, the trend of e-cigarette use among adolescents demonstrated pronounced sex-related differences^28,29^. Since the first national survey on e-cigarettes (TECMA 2016), the prevalence has remained substantially higher among males, reaching approximately 23.5% in 2022. Although the prevalence among females remained lower than that of males, it increased to 6.2% in 2022, representing a twofold rise from the baseline of 3.1% in 2016. This disparity is further supported by findings from multivariable analyses, which showed that the AOR of e-cigarette use was approximately four times higher among males than females. These findings align with global evidence in nearly all countries, where males exhibit a higher prevalence of e-cigarette use than females. In a systematic review of e-cigarette use among adolescents in Southeast Asia, seven out of ten studies identified male gender as a significant risk factor for e-cigarette use among adolescents^30^. Similarly, a multi-country analysis across 68 nations reported that among youth aged 12–16 years, the prevalence of e-cigarette use averaged 11.7% for males compared to 6.6% for females^22^. In this context, the 2022 prevalence of e-cigarette use among Malaysian male adolescents (23.5%) placed the country at the higher end of global estimates, while the corresponding rate among females (6.2%) remained comparatively moderate. Nonetheless, the doubling in prevalence among females highlights a concerning upward trend and a gradual narrowing of the sex gap in adolescent e-cigarette use in Malaysia.

Age-related differences in e-cigarette use among adolescents in Malaysia reveal a consistent pattern, whereby older adolescents are more likely to use e-cigarettes than their younger counterparts. From 2016 to 2022, although the prevalence did not increase markedly within specific age groups, the overall pattern indicated an upward trend in e-cigarette use with increasing age. This pattern is further supported by logistic regression analyses, which demonstrated a strengthening association between age and e-cigarette use from 2017 to 2022. A similar age gradient has been observed globally. In the United States, for example, a study among youths aged 13–18 years reported that the prevalence of current e-cigarette use peaked in the age group 16–18 years, approximately 30% in 2019, and remained consistently higher among older adolescents compared to younger ones^31^. Likewise, a global study involving 68 countries found that older youths (12–16 years) were significantly more likely to use e-cigarettes than their younger peers^22^. Malaysia’s pattern, wherein older adolescents demonstrate higher prevalence and risk of e-cigarette use, therefore aligns with international findings. These observations underscore the importance of designing prevention and intervention strategies that target not only early adolescents but also focus on mid- to late-adolescent groups, where initiation and uptake are more likely to occur.

This study shows that parental smoking status was associated with adolescents’ e-cigarette use in the NHMS 2022, although this association was not observed in the earlier 2017 survey. A similar pattern was also identified for parental e-cigarette use. Globally, adolescents with parents or family members who smoke, consistently show a significantly higher risk of e-cigarette use, a relationship supported by evidence across multiple regions. A meta-analysis of 21 studies found that having any family member who smokes increased the odds of adolescent e-cigarette use (OR=1.47), while the odds associated specifically with parental smoking were 1.41. In Southeast Asia, a systematic review similarly concluded that parental influence, including parental smoking, is a key socio-ecological factor associated with higher e-cigarette use among adolescents^30^, indicating that this parental effect persists across diverse cultural settings. Parental smoking or e-cigarette use may contribute to adolescent uptake by normalizing nicotine use within the household, lowering adolescents’ perceived harm, and increasing access to nicotine products.

In this trend analysis, Malaysia consistently demonstrates ethnic disparities in adolescent e-cigarette use, with Malays and the ‘other ethnicities’ exhibiting higher prevalence, a pattern that persists across TECMA and NHMS survey waves amid an overall rise in national prevalence. These persistent ethnic differences suggest that population-level prevention strategies alone may be insufficient. Targeted interventions that are culturally appropriate and context-specific, particularly for Malay adolescents, may be necessary to address the higher uptake of e-cigarettes observed in this group.

### Strengths and limitations

This study has several strengths. The analysis was based on nationally representative surveys with large sample sizes and high response rates. The use of standardized questionnaires adapted from the Global Youth Tobacco Survey, along with rigorous sampling and analytical procedures, enhances the reliability and generalizability of the findings to school-going adolescents in Malaysia.

Several limitations should also be considered. First, the cross-sectional nature of the data prevents causal interpretation of the associations observed and does not allow determination of the temporal sequence between exposure and outcome. Second, all measures relied on self-reported data, which may be subject to recall or social desirability bias, particularly among adolescents who report tobacco use. Third, the surveys included only school-going adolescents; therefore, the findings may not be generalizable to out-of-school youths. Fourth, the study describes changes across survey waves but does not formally test temporal trends using pooled data; therefore, the findings should be interpreted as cross-survey comparisons rather than a formal time-trend analysis. Finally, defining current e-cigarette use as any use within the past 30 days may classify occasional or experimental users together with more regular users.

## CONCLUSIONS

The prevalence of e-cigarette use among adolescents has substantially increased over the last decade in Malaysia. Although the patterns of associated sociodemographic factors remain generally consistent, the strength of these associations has changed for some factors over the study periods. These findings highlight the need for continued public health efforts and appropriate regulatory strategies to address e-cigarette use among adolescents in Malaysia.

## Supplementary Material



## Data Availability

The data supporting this research are available from the authors on reasonable request.
