# Supplementary material for: Trends in e-cigarette use among adolescents in Malaysia, 2016–2022: A secondary dataset analysis of national surveys
Source: Tob Induc Dis. 2026 May 26;24:10.18332/tid/219767. doi: 10.18332/tid/219767 (PMC13205650; doi:10.18332/tid/219767)
Supplement: Supplementary file 1 [file TID-24-72-s1.pdf]

Supplementary Table 1. Crude odds ratio of the association between factors and current e-cigarette use among school-going adolescents aged 13–17 years in Malaysia using complex sample univariable logistic regression, by survey wave (NHMS 2017, NHMS 2022) (analytic sample: 27,497; 33,523).

	2017			2022		
**Variables**	**Crude Odds Ratio**	**95% CI**		**Crude Odds Ratio**	**95% CI**	
		**Lower**	**Upper**		**Lower**	**Upper**
**Sex [ref: female]**						
Male	7.05	5.79	8.58	4.65	4.14	5.22
**Age (Form) [ref: 13 (Form 1)]**						
14 (Form 2)	1.12	0.85	1.47	1.22	1.03	1.45
15 (Form 3)	1.2	0.85	1.68	1.5	1.25	1.79
16 (Form 4)	1.27	0.95	1.69	1.75	1.37	2.22
17 (Form 5)	1.25	0.89	1.76	2	1.58	2.47
**Ethnicity [ref: Chinese]**						
Malays	2.42	1.84	3.18	3.71	3	4.58
Indians	2.59	1.56	4.3	2.18	1.6	2.92
Others	3.45	2.54	4.67	4.62	3.68	5.8
**Smoking Status [ref: non-smoker]**						
Smoker	25.96	22.39	30.11	63.34	55.26	72.62
**Parents smoking status [ref: non-smoker]**						
Smoker	1.95	1.7	2.23	1.87	1.71	2.05
**Parents ecig user [ref: non-user]**						
Ecig user	3.74	3.09	4.51	2.03	1.84	2.24

© 2026 Yusoff M.F.M. et al.
